# A Secure Mutual Batch Authentication Scheme for Patient Data Privacy Preserving in WBAN

**DOI:** 10.3390/s19071608

**Published:** 2019-04-03

**Authors:** Martin Konan, Wenyong Wang

**Affiliations:** Department of Computer Science and Engineering, University of Electronic Science and Technology of China (UESTC), Chengdu 611731, China; wangwy@uestc.edu.cn

**Keywords:** batch authentication, certificate less scheme, data aggregation, data privacy, elliptic curve cryptography (ECC), wireless body area networks (WBAN)

## Abstract

The current advances in cloud-based services have significantly enhanced individual satisfaction in numerous modern life areas. Particularly, the recent spectacular innovations in the wireless body area networks (WBAN) domain have made e-Care services rise as a promising application field, which definitely improves the quality of the medical system. However, the forwarded data from the limited connectivity range of WBAN via a smart device (e.g., smartphone) to the application provider (AP) should be secured from an unapproved access and alteration (attacker) that could prompt catastrophic consequences. Therefore, several schemes have been proposed to guarantee data integrity and privacy during their transmission between the client/controller (C) and the AP. Thereby, numerous effective cryptosystem solutions based on a bilinear pairing approach are available in the literature to address the mentioned security issues. Unfortunately, the related solution presents security shortcomings, where AP can with ease impersonate a given C. Hence, this existing scheme cannot fully guarantee C’s data privacy and integrity. Therefore, we propose our contribution to address this data security issue (impersonation) through a secured and efficient remote batch authentication scheme that genuinely ascertains the identity of C and AP. Practically, the proposed cryptosystem is based on an efficient combination of elliptical curve cryptography (ECC) and bilinear pairing schemes. Furthermore, our proposed solution reduces the communication and computational costs by providing an efficient data aggregation and batch authentication for limited device’s resources in WBAN. These additional features (data aggregation and batch authentication) are the core improvements of our scheme that have great merit for limited energy environments like WBAN.

## 1. Introduction

Recall that recent innovations are done in the wireless sensor network (WSN), which have cleared the route for smart sensors that can be embedded on the human body to monitor glucose and respiratory rate, for example [1,2,3,4,5]. This interconnectedness of various advanced handheld gadgets worn or embedded in human systems is referred to as a wireless body area network (WBAN). WBAN commonly incorporates a cell phone at the client’s side that acts as a center point/controller, obtaining the client’s information and transferring it to a remote server or Application Provider (AP).

Despite the fact that WBAN has enhanced the e-Care administration system, the security and privacy of client’s data remain a tremendous challenge to address [3,4,5,6,7,8,9,10,11,12,13,14,15,16]. For instance, a client should know about the AP dealing with his/her related information before asking for further data processing (data accountability issue). Therefore, there is a paramount need for the client, as well as the e-Care system agents (doctors, medical attendants, etc.), to authenticate each other to preserve data confidentiality. Thereby the physician can ascertain the correctness of physiological information diagnostic that may have cataclysmic consequences on a patient in case of wrong authentication. Hence developing a new cryptosystem that ensures integrity, authentication, accountability, accessibility, non-repudiation, and secrecy is considered a hot topic by the information security research community [3,4,5,6,7,8,9,10,11,12,13,14,15,16]. A cryptosystem that provides mutual authentication scheme between the controller (C) and AP is crucial in order to preserve data security. For this reason, several valuable contributions have been introduced to securely transmit data from a given C to AP [3,4,5,6,7,8,9,10,11,12,13,14,15,16,17,18,19,20,21,22,23]. Note that those existing authentication schemes use different approaches that can be classified as: (i) physiological value based, (ii) channel based, (iii) proximity based, and (iv) cryptographic based [4].

Our proposed solution uses the cryptographic technique tools. Likewise, various cryptosystem schemes are presented among the research community [3,4,5,6,7,8,9,10,11,12,13,14,15,16]. However, the traditional asymmetric encryption (public key infrastructure PKI) technique that acts as primary solution to provide security is an inefficient option for optimized lightweight cryptosystem design in constrained resource environments (WBAN). This reason is due to the inherent PKI database administration issues, i.e., capacity, data transfer, and annulment and confirmation of certificates. To address this certificate administration issue, researchers [5] presented a new idea of an identity-based encryption (IBE) cryptosystem. This novel identity-based public key cryptographic (IB-PKC) model allows the client’s secret key to be an element of his real identity, which is generated by a trusted outsider called a private key generator (PKG). In this way, a genuine public key does not require certificates [6]. However, the fact that PKG exclusively generates private keys for clients raises a security shortcoming known as a key escrow issue. With a specific end goal to tackle this mentioned issue, researchers in [7] presented a certificate-less (CL) cryptography scheme, generally denoted as CL-PKC.

**Figure 1:**
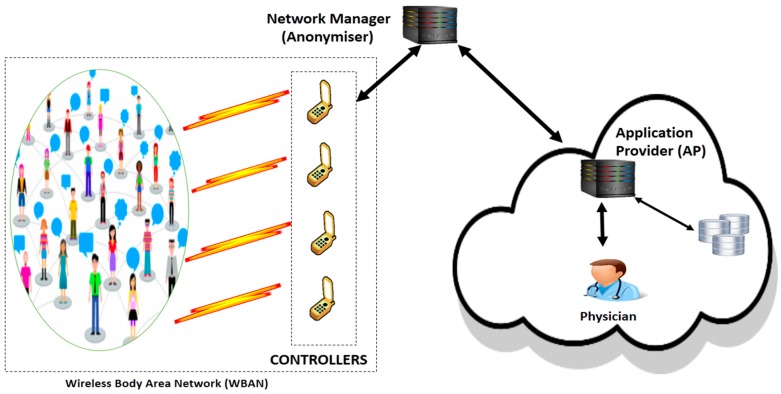
Proposed scenario.

Recall that WBAN is based on remote wireless sensors that can transmit only within short ranges, with low handling power and energy [7]. To address this short communication range issue of medical records to a longer distance, a smart intermediate mobile device (e.g., a cell phone, also named a controller) is used inside the WBAN’s communication range (refer to Figure 1). Therefore, all cryptosystems with high computation cost to guarantee a high data security level are inappropriate. Hence various efficient authentication models other than the traditional PKI are presented in the literature [3,4,8,9,10,11,12,13,14,15,16,18,20]. Security insurance issues have started to draw escalated consideration among researchers and, lately, authors in [4] raised the shortcoming of an impersonation attack in a related scheme [8]. This security shortcoming resulted from saving the encryption and decryption keys on an unreliable AP database, and therefore introduced a novel secured authentication solution [4]. Furthermore, authors in [9] also proved that the existing cryptosystem [8] could not address the well-known stolen verifier–table attack. Thus, they proposed an authentication protocol based on elliptic curve cryptography (ECC) [9], notwithstanding that researchers in [10] proved that a related model [9] could not provide genuine anonymous data, while client’s pseudo attributes could be utilized to track the corresponding clients. Therefore, an improved cryptosystem based on a user’s identity was presented [10] to securely authenticate the different entities using bilinear pairing. 

Due to the openness and mobility of WBAN, the transmission must be anonymous and unlinkable as well. In this way, authors in [11] designed a scheme that allowed sensor nodes appended in a patient’s body to authenticate with a local server/hub node and establish a session key in an anonymous and unlinkable way. This scheme [11] was proposed to as efficient as possible, by using only two types of operations: the cryptographic hash function and the exclusive OR operation (XOR). Likewise, Aneesh and Deepthi [12] presented a hybrid anonymous authentication and key agreement scheme, which was an improvement based on Li et al.’s scheme [11] using the physiological signal to overcome the node impersonation issue [8]. In this proposed solution [12], authors provided additional security features to effectively address the node impersonation and key escrow issues [6,8]. Aneesh and Deepthi [12] highlighted some security shortcomings in Li et al.’s scheme [11] and used physiological signals to resolve them. This made the proposed scheme a hybrid scheme. Practically, the related schemes [11,12] security proofs used Burrows–Abadi–Needham (BAN) logic and the Automated Validation of Internet Security Protocols and Applications (AVISPA). However, the use of physiological signals implies that all sensors nodes measure the same physiological signal and introduce additional costs for the collecting and transforming of data, as well as maintaining all sensors synchronized. 

Therefore, Marko et al.’s model [13] showed that the schemes [11,12] fell short of their goals, and, in fact, did not provide untraceability of the communicating sensor nodes. Based on that, the goal was to provide a solution [13] with anonymous participants without session linkability/ traceability. This new scheme achieved the untraceability property, while retaining computational complexity and reducing the communication costs. By achieving untraceability, the proposed solution could be a good candidate to improve Koya et al.’s scheme [12]. However, this scheme increased the required storage space. Furthermore, the security proof of the new scheme was discussed informally with some well-known attacks and was formally provided using the BAN logic, the AVISPA, and Scyther tool.

A new view of achieving user anonymity property has been introduced by using a Smartcard instead of traditional authentication scheme method to address the security and privacy issues in wireless multimedia sensor networks (WMSNs). Thereby, Ashok et al.’s [14] reviewed Li et al.’s scheme [11] and proved that their solution was still vulnerable to privileged-insider attack and sensor node capture attack, and failed to provide user anonymity properties. In order to address these security shortcomings found in Li et al.’s scheme [11], they proposed a secure biometrics-based user authentication scheme in WMSNs using a smartcard. This new scheme has been rigorously proven secure against possible known attacks and efficient in computation and communication as compared to Li et al.’s scheme [11]. As a further matter, a fresh approach has been tackled with the emergence of quantum computers to achieve the anonymity property. So far, most of the above-mentioned solutions are based on bilinear pairing and an elliptic curve cryptosystem. However, their security is based on the discrete logarithm on the elliptic curve, which has been proven to be limited by the development of quantum computers. To address the issue, Rui et al. [15] presented a new lightweight anonymous handover authentication (AHA) scheme based on the Number Theory Research Unit (NTRU) public key cryptosystem for wireless networks. Security analysis and experimental results showed that this scheme achieved mutual authentication with a greater security level to address known attacks. The advantages of the proposed scheme are the low computation cost, high efficiency, and ease of implementation as compared to related works like [11,12]. However, the disadvantage is that this scheme [15] cannot predict the misbehaving nodes and avoid the collusion attacks due to the lack of trust and reputation evaluation mechanism. Its correctness is only based on the certification results of both parties. Therefore, this proposed solution [15] is only suitable for the scenario of a single authentication model with a few participants.

In this paper, our contribution will be first to identify and propose a certificate-less mutual authentication scheme that addresses the impersonation issue in the related works [8,9,10]. Second, we design a lightweight cryptographic algorithm using an effective combination of ECC and bilinear pairings operation for limited devices in WBAN. Furthermore, our proposed solution is more efficient than the existing works [8,9,10,15] by providing a batch authentication process that reduces considerably the computation and communication costs for constrained resource devices in WBAN.

The rest of this work is sectioned as follows. Section 2 presents the background work, while Section 3 gives the detailed design of the proposed solution. Section 4 analyzes and evaluates the performance and security level of the proposed contribution. Then, we end this work in Section 5.

## 2. Proposed Solution Construction

### 2.1. Preliminaries

#### 2.1.1. Elliptic Curve Cryptography (ECC)

ECC is an asymmetric key encryption scheme based on elliptic curve theory that generates faster, smaller, and efficient cryptosystem keys. It was introduced by Koblitz [24] and Miller [25]. A fixed curve E over a field K can be described in a non-homogeneous manner by the following equation (Weierstrass equation) [26]:

(1) $$y^{2}+a_{1}\mathrm{xy}+a_{3}y=x^{3}+a_{2}x^{2}+a_{4}x+a_{6}$$

where $a_{1},a_{2},a_{3},a_{4},a_{6}\in K$ and Δ≠0, and where Δ is the discriminant of E and is defined as follows:

(2) $$\left\{ \begin{array}{c} \Delta =-d_{2}{}^{2}d_{8}-8d_{4}{}^{3}-27d_{6}{}^{2}+9d_{2}d_{4}d_{6}\\ d_{2}=a_{1}{}^{2}+4a_{2};\;d_{4}=2a_{4}+a_{1}a_{3};\;d_{6}=a_{3}{}^{2}+4a_{6}\\ d_{8}=a_{1}{}^{2}a_{6}+4a_{2}a_{6}-a_{1}a_{3}a_{4}+a_{2}a_{3}{}^{2}-a_{4}{}^{2} \end{array}\right.$$

Based on the literature review, ECC can provide a strong secured cryptosystem with a 164-bit key, while others cryptographic schemes require a 1024-bit key. Therefore, ECC is more appropriate to achieve the desired security level with the lowest computation power cost and device battery usage. Thus, it is a suitable and efficient solution for limited mobile device applications. The security advantage of ECC lies in its competitive short security key size and the strong assumption to solve the elliptic curve discrete logarithm problem (ECDLP).

#### 2.1.2. Bilinear Pairings

Bilinear maps explained in [27] can be presented as follows: Let two cyclic groups $E_{1}$ (additive) and $E_{2}$ (multiplicative) of order p (prime number). Let *g* be a generator of $E_{1}$, and e a bilinear mapping; then,

e: $E_{1}$ × $E_{1}$ → $E_{2}$. The bilinear mapping e satisfies these properties:

♦ Bilinearity: $\forall \;A,\;B\in E_{1},\;\forall \;d,\;f\in {\mathbb{Z}}_{p}^{*},$
     $e\left(\mathrm{dA},\;\mathrm{fB}\right)=e{\left(A,\;B\right)}^{\mathrm{df}}$
♦ Non-Degeneracy: ∃ A, B ∈ E_1_ such that e (A, B) ≠ 1, and 1 is the identity element of E_2_.
♦ Computation: For any A, B ∈ $E_{1}$, we have an efficient algorithm to compute e.

Recall that a group that has such a mapping e is defined as a bilinear group on which the Decisional Diffie–Hellman issue can be easily solved, while the Computational Diffie–Hellman (CDH) issue is considered very hard. Therefore, our proposed solution is based on the below security computational assumptions.

### 2.2. Security Assumption

We propose an efficient mutual batch authentication solution relying on strong security computation assumptions.

**Problem** **1:** *Consider a multiplicative cyclic group G of order p, with generator g. A probabilistic polynomial–time adversary has a negligible chance to compute* $g^{\mathrm{ab}}$ *, from* g *,* $g^{a}$ *,* $g^{b}$ *for random* $a,\;b\in Z_{p}^{*}$ *.*

**Problem** **2:** *Elliptic curve discrete logarithm problem (ECDLP). Let E be elliptic curve over a finite field K. Suppose points* P, Q∈E(K) *, it is difficult to determine k such that* Q=[k]P *, with* Q∈E(K) *.*

**Figure 2:**
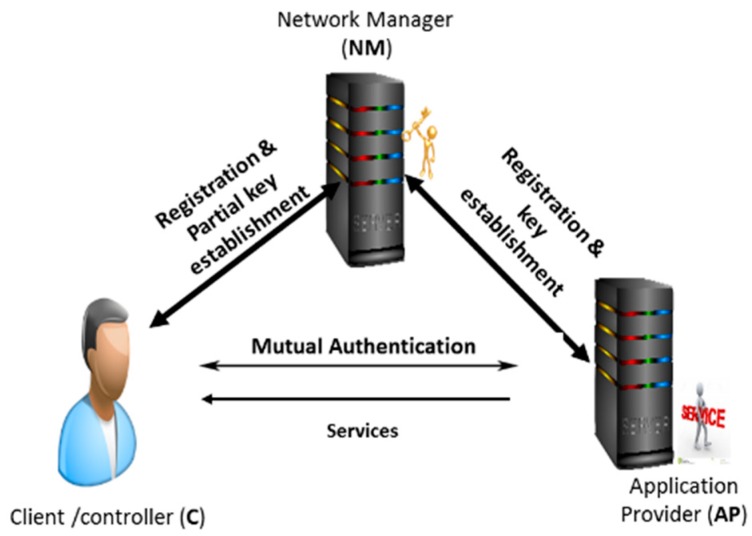
Mutual authentication overview.

Here, we propose an architecture that is depicted by Figure 1, which is comprised of the WBAN, the controller/client (C), the network manager (NM), and the application provider (AP). WBAN is a particular environment where a sensor is organized to work self-sufficiently by connecting to different medicinal sensors, situated inside and outside of a human body system. The sensors transmit medical information to a remote AP server via C. Therefore, in our proposed solution we focus on the mutual authentication between C and AP to guarantee data integrity and confidentiality. The main steps in this mutual authentication scheme, i.e., initialization, registration, and authentication between C and AP [4,7,28,29], are done via a reliable outsider NM as depicted in Figure 2. In this scenario, C and AP register with NM to get the different partial cryptographic keys. Thereby, NM assumes the duty of the key generator center (KGC). Contrary to related works in the literature, where NM is completely trustworthy, we assume in this paper that NM could be curious and dishonest. Therefore, C and AP register with NM to obtain not the full key but partial cryptographic key parameters for stronger data privacy protection. In order to address the various attacks (passive or active) [30], our scheme provides the following security requirements:

(i) Mutual authentication: It will ensure that exclusive genuine and approved C gets access privileges from AP and similarly just approved AP will receive and process data from C.
(ii) Anonymity: This prerequisite guarantees that an attacker does not have access to the genuine partaker’s identity (C and AP) in their identification procedure.
(iii) Unlinkability: This condition guarantees that an attacker cannot interface C’s identity to a particular session while asking for computations from AP.
(iv) Furthermore, our proposed solution provides resilience to replay and impersonation attack. Further, used keys cannot be recovered by an attacker, and our solution does not use verification table.

Recall that the principal objective of this work is to design an efficient batch mutual certificate-less authentication scheme between C and AP that ascertains their identity in the communication process. Thereby a passive attacker (eavesdropper) should have a slight chance to impersonate either C or AP. Further, by providing anonymity, we upgrade the client’s privacy protection since the unlinkability property is guaranteed.

### 2.3. Related Work

Wang and Zhang proposed a new anonymous authentication scheme for WBAN [7] to overcome the security weaknesses of Zhao’s model [9]. We can describe the different steps, i.e., Initialization, Registration, and Authentication phases of their model as follows.

♦ **Initialization phase:** It mainly consists of generating keys and system parameters and it is done by the NM shown below.
  (i) NM computes a large prime number q, two groups $G_{1}$, $G_{2}$, a pairing map
    $e:G_{1}\times G_{1}\to G_{2}.$
  (ii) NM selects two secured hashing maps h and H, where h:{0, 1}*→Zq and $H:\left\{0,\;1\right\}*\to G_{1}$.
  (iii) NM generates randomly a number $s_{\mathrm{NM}}\in Z_{q}$ as its secret key and compute $Q=s_{\mathrm{NM}}P$ as its public key.
  (iv) Finally, NM provides as public parameters $\mathrm{params}=\left\{q,{\;G}_{1},G_{2},e,P,\;h,\;H,Q_{\mathrm{NM}}\right\}$.
♦ **Registration phase:** It is during this step that C and AP get registered with NM to get their different partial private key.
  (i) The entity C/AP transmits his identity ${\mathrm{ID}}_{O}$ to NM.
  (ii) With ${\mathrm{ID}}_{O}$, NM computes the partial secret key $S_{O}=s_{\mathrm{NM}}Q_{O}$, where $Q_{O}=H\left({\mathrm{ID}}_{O}\right)$. NM then sends secret key $S_{O}$ to *O* through a secure channel.
  (iii) C/AP secretly stores its partial private key $S_{O}$.
♦ **Authentication phase:** At this phase *C*/AP mutually authenticates each other and computes secured keys to encrypt patient records as follows:
  (i) C generates a random number $r_{C}\in Z_{q}{}^{*}$, calculates $Q_{\mathrm{AP}}=H\;\left({\mathrm{ID}}_{\mathrm{AP}}\right),Q_{C}=H\left({\mathrm{ID}}_{C}\right)$, $R_{C}=r_{C}{\;Q}_{C},K_{C}=e\;\left(S_{C},r_{C}Q_{\mathrm{AP}}\right),{\;\mathrm{and}\;\mathrm{Auth}}_{C}=E_{\mathrm{KC}}\left({\mathrm{ID}}_{C}\left|\left|T_{C}\right|\right|R_{C}\right)$. With, $T_{C}$ the current timestamp. Then, C sends a message $M_{1}=\left\{R_{C},T_{C},{\;\mathrm{Auth}}_{C}\right\}$ to AP.
  (ii) With $M_{1}=\left\{R_{C},T_{C},{\;\mathrm{Auth}}_{C}\right\}$, AP verifies the freshness of $T_{C}$ and rejects if it is not fresh. AP computes $K_{\mathrm{AP}}=e\;\left(S_{\mathrm{AP}},\;R_{C}\right)$ and gets $\left({\mathrm{ID}}_{C}\left|\left|T_{C}\right|\right|R_{C}\right)$ by decrypting ${\mathrm{Auth}}_{C}$. Then AP compares if $T_{C}$ and the decrypted one are equal. If not matching, AP cancels the access process. Else, AP computes randomly a number $r_{\mathrm{AP}}$ ∈ $Z_{q}{}^{*}$ and computes $Q_{C},Q_{\mathrm{AP}},\;R_{\mathrm{AP}}=r_{\mathrm{AP}}Q_{C},\;L_{\mathrm{AP}}=r_{\mathrm{AP}}R_{C}$ ${\mathrm{Auth}}_{\mathrm{AP}}=h\left(T_{C}\left|\left|R_{C}\right|\right|R_{\mathrm{AP}}\left|\left|K_{\mathrm{AP}}\right|\right|L_{\mathrm{AP}}\right)$ session key ${\mathrm{sk}}_{\mathrm{AP}}=h\left(T_{C}\left|\left|R_{C}\right|\right|T_{\mathrm{AP}}\left|\left|R_{\mathrm{AP}}\right|\right|L_{\mathrm{AP}}\right)$, where $T_{\mathrm{AP}}$ is the actual time stamp. Then, AP transfers message $M_{2}=\left\{R_{\mathrm{AP}},\;T_{\mathrm{AP}},\;{\mathrm{Auth}}_{\mathrm{AP}}\right\}$ to C.
  (iii) Receiving $M_{2}$, C verifies the freshness of $T_{\mathrm{AP}}$. If not, C stops the access demand. Otherwise, C computes $L_{C}=r_{C}R_{\mathrm{AP}}$, and checks the correctness of the equation ${\mathrm{Auth}}_{\mathrm{AP}}=h\left(T_{C}\left|\left|R_{C}\right|\right|R_{\mathrm{AP}}\left|\left|K_{\mathrm{AP}}\right|\right|L_{\mathrm{AP}}\right)$. Then, C computes session key ${\mathrm{Sk}}_{C}=h\left(T_{C}\left|\left|R_{C}\right|\right|T_{\mathrm{AP}}\left|\left|R_{C}\right|\right|L_{C}\right),$ else the answer is rejected.

### 2.4. The Security Shortcoming

Based on the above description of Wang and Zhang’s model (WZ) [7], a given AP can simulate a client ($K_{C}$ = $K_{\mathrm{AB}}$). Therefore, a malicious AP could impersonate C as following: the attacker picks $r_{C}\in_{R}{\;Z}_{q}{}^{*}$, sets $Q_{C}=H_{1}\left({\mathrm{ID}}_{C}\right),\;\mathrm{and}$ computes $R_{C}=r_{C}Q_{C}$. Thereby the attacker can compute his/her own $K^{*}{}_{\mathrm{AB}}=e\;\left(S_{\mathrm{AP}},\;R_{C}\right)=K_{C}$ and then generate a correct login $\left\{{M\;}_{1}=R_{C},{\mathrm{Auth}}_{C},T\right\}$, and ${\mathrm{Auth}}_{C}=h\;\left(T\left|\left|K_{C}\right|\right|R_{C}\right)$. This security weakness is due to the absence of an authenticator in the generated $K_{C}$. Therefore, the WZ solution presents a security shortcoming during the C/AP authentication process. To address this shortcoming, authors in [29] proposed an effective remote identity validation scheme. Based on their experiment results [29], this existing solution can provide a malignant insider security, as well as reduce running time of C by 51% when contrasted with Wang and Zhang’s model [7]. However, those related works do not provide data aggregation and batch mutual identity validation processes to reinforce the data privacy protection.

## 3. Proposed Solution

Authentication issues related to patients in the e-Care system have begun to draw intense attention in the literature [31]. Therefore, we present in this section our contribution by designing a strong mutual certificate-less authentication scheme between C and AP. The Table 1 summarizes the different abbreviations used in this paper.

Our proposed solution satisfies the following security requirements to guarantee that an attacker cannot impersonate either AP or C and modifies the transmitted data (integrity of data and privacy of the client C assurance).

(1) Subscriber authentication: AP should confirm the various C’s identity to guarantee their authenticity.
(2) Provider validation: A client C is permitted to verify the different AP’s identity it visits to keep away from potential forgery and various malevolent attacks.
(3) Key generation: A different encryption key is generated each time C and AP initiate a session to ensure the protection of the transferred data.
(4) Anonymous Client: Apart NM, the client C is unknown and its operations are unlinkable to anybody including the AP.

### 3.1. Security System Settings

NM sets the entire system (sets parameters) and computes the partial secret keys by running the following steps based on elliptic curve E/Fq and random generator *P* for $G_{1}$ (cyclic additive group).

NM randomly selects a number $S_{NM}\in Z_{q}{}^{*}$ as master private key and calculates his related public key $PK_{NM}=S_{NM}P$.

Then, NM picks below hashing mappings:

$H_{1}:{\left\{0,1\right\}}^{l}\times G_{1}{}^{2}\to Z_{q}{}^{*},$$H_{2}:G_{1}{}^{2}\times {\left\{0,1\right\}}^{2l}\to Z_{q}{}^{*}$, with l and k specifying identity’s length and size in $Z_{q}{}^{*}$. NM publishes system public parameters $\mathrm{params}=\left\{P_{\mathrm{NM}},{\;H}_{1},H_{2},P,\;E/\mathrm{Fq},{\;G}_{1}\right\}$

### 3.2. Registration Phase

We use data privacy preserving tools relying on pseudonyms. C usually has enough storage backup to handle a huge quantity of preloaded pseudonyms from NM. An effective work [32] addresses the data backup issue related to preload anonymous cryptosystem keys (pseudonyms). In this paper, the proposed scheme requires a pool of pseudonyms with short live times (based on expiry date), where the memory consumption is limited to the related work’s results [32]. This approach is used by several existing models and has been proven efficient, especially for wireless environments.

The NM then provides a list of pseudonyms (pseudo identity/pseudo-ID) for C and generates partially the secret keys for both AP and C, respectively, like in [28] with some modifications in the registration phase.

#### 3.2.1. The Client C Registration 

C with its identity ${\mathrm{ID}}_{c}\in {\left\{0,1\right\}}^{l}$ picks randomly $x_{C}\in Z_{q}{}^{*}$ as its secret value, computes its public key as ${\mathrm{PK}}_{C}=x_{C}\;P,$ then C transfers ${\mathrm{ID}}_{c}$, ${\mathrm{PK}}_{C}$ to NM that first verifies the C’s identity validity. If ${\mathrm{ID}}_{c}$ is genuine, then NM randomly picks a family of unlinkable pseudo-ID:

${\mathrm{PID}}_{C}=\left\{{\mathrm{pid}}_{c1},{\mathrm{pid}}_{c2},\ldots \right\}$ With a specified-lived valid period. Then NM generates a secret random number $r_{c}\in Z_{q}{}^{*}$, and computes $P_{C}=r_{c}P$.

For each pseudo-ID ${\mathrm{pid}}_{\mathrm{cj}}\in {\mathrm{PID}}_{C}$, NM computes the secret value $S_{C}=(r_{c}+H_{1}\left({\mathrm{pid}}_{\mathrm{cj}},{\mathrm{PK}}_{C},P_{C}\right)S_{\mathrm{NM}})\;\mathrm{mod}\;q$ and sets C’s partial private key as $S_{\mathrm{NM}}.H_{1}\left(S_{C}\right)$. Then NM sends securely all the tuples ($S_{\mathrm{NM}}.H_{1}\left(S_{C}\right),{\;P}_{C},S_{C}P$) back to C. Thereby C can ascertain the validation of its partial secret key by verifying if the equation $S_{C}P=P_{C}+H_{1}\left({\mathrm{pid}}_{\mathrm{cj}},{\mathrm{PK}}_{C},P_{C}\right){\mathrm{PK}}_{\mathrm{NM}}$ holds for each ${\mathrm{pid}}_{\mathrm{cj}}\in {\mathrm{PID}}_{C}$. Therefore, the full private key of C is generated and known by C only with the value equal to $\left(x_{C},S_{\mathrm{NM}}.H_{1}\left(S_{C}\right)\right)$. Doing so, C can change its pseudo-ID $\left({\mathrm{pid}}_{\mathrm{cj}}\right)$, in the valid time period to achieve identity privacy in mutual authentication process with AP.

#### 3.2.2. Application Provider AP Registration

Similarly, AP and its identity ${\mathrm{ID}}_{\mathrm{AP}}\in {\left\{0,1\right\}}^{l}$ sets $x_{\mathrm{AP}}\in Z_{q}{}^{*}$ as secret key, computes its public key as ${\mathrm{PK}}_{\mathrm{AP}}=x_{\mathrm{AP}}\;P,$ then transfers ${\mathrm{ID}}_{\mathrm{AP}},{\;\mathrm{PK}}_{\mathrm{AP}}$ to NM. Again, NM chooses random number $r_{\mathrm{AP}}\in Z_{q}{}^{*}$, computes $P_{\mathrm{AP}}=r_{\mathrm{AP}}P$, $S_{\mathrm{AP}}=(r_{\mathrm{AP}+}H_{1}\left({\mathrm{ID}}_{\mathrm{AP}},{\mathrm{PK}}_{\mathrm{AP}},P_{\mathrm{AP}}\right)S_{\mathrm{NM}})\mathrm{mod}\;q.$

Then NM sets as partial private key $S_{\mathrm{NM}}.H_{1}\left(S_{\mathrm{AP}}\right)$ for AP and secretly (e.g., using a secure transmission protocol) sends ($S_{\mathrm{NM}}.H_{1}\left(S_{\mathrm{AP}}\right)$, $P_{\mathrm{AP}},{\;S}_{\mathrm{AP}}P$) to AP. In order to verify the correctness of $S_{\mathrm{AP}}$, AP verifies if $S_{\mathrm{AP}}P=P_{\mathrm{AP}}+H_{1}\left({\mathrm{ID}}_{\mathrm{AP}},\;{\mathrm{PK}}_{\mathrm{AP}},P_{\mathrm{AP}}\right){\mathrm{PK}}_{\mathrm{NM}}$ holds and keeps this value. Likewise, AP sets its full private key as $(x_{\mathrm{AP}},S_{\mathrm{NM}}.H_{1}\left(S_{\mathrm{AP}}\right)$).

In the above registration process, NM appends Expire Date into each ${\mathrm{pid}}_{\mathrm{cj}}\in {\mathrm{PID}}_{C}$. The validity of the partial private keys is then set before a specific date. Thus, the partial secret keys are automatically removed after that date, and fresh partial secret keys with new validity date are generated by NM. This key management approach securely can be given to C (even damaged, hacked, or stolen) without compromising seriously the system security. More, we avoid key and certificate management like in the traditional PKI environment and provide user revocation.

### 3.3. Authentication Phase

**Figure 3:**
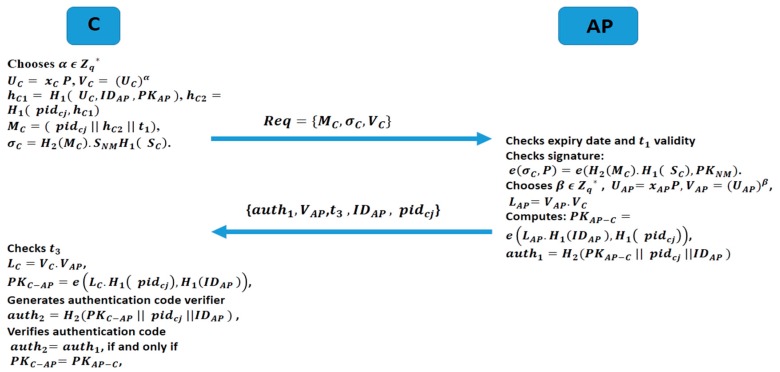
C and AP authentication process overview.

The focus here is to provide a secured mutual authentication scheme between C and AP that ascertains their identity to guarantee the physiological data’s privacy during their communication process. Below are the different steps involved in this authentication process between C and AP depicted by the Figure 3:

(1). C picks a random unused pseudo-ID (${\mathrm{pid}}_{\mathrm{cj}}$) and its corresponding partial private key $S_{\mathrm{NM}}.H_{1}\left(S_{C}\right)$. Then C chooses randomly $\alpha \;\epsilon {\;Z}_{q}{}^{*}$ and compute $U_{C}=x_{C}\;P$, and a session verifier $V_{C}={\left(U_{C}\right)}^{\alpha }$.
(2). C computes $h_{C1}=H_{1}\left(U_{C},{\mathrm{ID}}_{\mathrm{AP}},\;{\mathrm{PK}}_{\mathrm{AP}}\right)$, $h_{C2}=H_{1}\left({\mathrm{pid}}_{\mathrm{cj}},h_{C1}\right)$ and composes message $M_{C}=\left({\mathrm{pid}}_{\mathrm{cj}}\left|\left|h_{C2}\right|\right|t_{1}\right)$.
(3). The client C computes a signature $\sigma_{C}=H_{2}\left(M_{C}\right).S_{\mathrm{NM}}H_{1}\left(S_{C}\right)$ and sends a request message to AP: $\mathrm{Req}=\left\{M_{C},\sigma_{C},V_{C}\right\}$ with Δt the valid transmission delay calculated by C.
(4). Upon receiving the request message (Req) at time $t_{2}$ from C, AP first verifies the expiry date in ${\mathrm{pid}}_{\mathrm{cj}}$. If the expiry date is valid, AP then checks the freshness of $t_{1}$ by verifying if $t_{2}-t_{1}\leq \Delta t.$ If $t_{1}$ is fresh, AP with the public parameters params, verifies the validity of C’s signature $\sigma_{C}$ by checking if the Equation (3) holds.
  (3) $$e\left(\sigma_{C},P\right)=e\left(H_{2}\left(M_{C}\right).H_{1}\left(S_{C}\right),{\mathrm{PK}}_{\mathrm{NM}}\right)$$
  ***Verification:***
  $$e\left(\sigma_{C},P\right)=e\left(H_{2}\left(M_{C}\right).S_{\mathrm{NM}}H_{1}\left(S_{C}\right),P\right)$$
  $$=e\left(H_{2}\left(M_{C}\right).H_{1}\left(S_{C}\right),S_{\mathrm{NM}}P\right)$$
  $$e\left(\sigma_{C},P\right)=e\left(H_{2}\left(M_{C}\right).H_{1}\left(S_{C}\right),{\mathrm{PK}}_{\mathrm{NM}}\right)$$
(5). AP selects randomly $\beta \;\epsilon {\;Z}_{q}{}^{*}$ and computes: $U_{\mathrm{AP}}=x_{\mathrm{AP}}P,$ $V_{\mathrm{AP}}={\left(U_{\mathrm{AP}}\right)}^{\beta }$ (session verifier), and $L_{\mathrm{AP}}=V_{\mathrm{AP}}.V_{C}$.
(6). Then AP computes a private session key ${\mathrm{PK}}_{\mathrm{AP}-C}=e\left(L_{\mathrm{AP}}.H_{1}\left({\mathrm{ID}}_{\mathrm{AP}}\right),H_{1}\left({\mathrm{pid}}_{\mathrm{cj}}\right)\right)$ and generates an authentication code ${\mathrm{auth}}_{1}=H_{2}\left({\mathrm{PK}}_{\mathrm{AP}-C}\left|\left|{\mathrm{pid}}_{\mathrm{cj}}\right|\right|{\mathrm{ID}}_{\mathrm{AP}}\right)$ and sends {${\mathrm{auth}}_{1},V_{\mathrm{AP}}$, $t_{3},\;{\mathrm{ID}}_{\mathrm{AP}},{\;\mathrm{pid}}_{\mathrm{cj}}$} to C.
(7). Upon receiving {${\mathrm{auth}}_{1},V_{\mathrm{AP}}$, $t_{3},\;{\mathrm{ID}}_{\mathrm{AP}},\;{\mathrm{pid}}_{\mathrm{cj}}$} at $t_{4}$ from AP, the client C verifies the freshness of $t_{3}$ by checking if $t_{4}-t_{3}\leq \Delta {}^{\prime }t$, with ∆${}^{\prime }t$ the valid transmission delay calculated by AP. If $t_{3}$ is fresh, C computes $L_{C}=V_{C}.V_{\mathrm{AP}}$, and a private symmetric session key with AP like: ${\mathrm{PK}}_{C-\mathrm{AP}}=e\left(L_{C}.H_{1}\left({\mathrm{pid}}_{\mathrm{cj}}\right),H_{1}\left({\mathrm{ID}}_{\mathrm{AP}}\right)\right).$ Furthermore, C generates an authentication verification ${\mathrm{auth}}_{2}=H_{2}\left({\mathrm{PK}}_{C-\mathrm{AP}}\left|\left|{\mathrm{pid}}_{\mathrm{cj}}\right|\right|{\mathrm{ID}}_{\mathrm{AP}}\right)$ code and compares with ${\mathrm{auth}}_{1}$. If ${\mathrm{auth}}_{2}={\mathrm{auth}}_{1}$, then C can ascertain the identity of AP as legitimate; otherwise, C stops the communication process with AP and reports it to NM. In this scenario C can verifies if ${\mathrm{auth}}_{2}={\mathrm{auth}}_{1}$ if and only if ${\mathrm{PK}}_{C-\mathrm{AP}}={\mathrm{PK}}_{\mathrm{AP}-C}$:
  $${\mathrm{PK}}_{C-\mathrm{AP}}=e\left(L_{C}.H_{1}\left({\mathrm{pid}}_{\mathrm{cj}}\right),H_{1}\left({\mathrm{ID}}_{\mathrm{AP}}\right)\right)$$
  $$=e\left(V_{C}.V_{\mathrm{AP}}.H_{1}\left({\mathrm{pid}}_{\mathrm{cj}}\right),H_{1}\left({\mathrm{ID}}_{\mathrm{AP}}\right)\right)$$
  $$=e\left(V_{\mathrm{AP}}.V_{C}.H_{1}\left({\mathrm{pid}}_{\mathrm{cj}}\right),H_{1}\left({\mathrm{ID}}_{\mathrm{AP}}\right)\right)$$
  $$=e\left(L_{\mathrm{AP}}.H_{1}\left({\mathrm{pid}}_{\mathrm{cj}}\right),H_{1}\left({\mathrm{ID}}_{\mathrm{AP}}\right)\right)$$
  $${\mathrm{PK}}_{C-\mathrm{AP}}={\mathrm{PK}}_{\mathrm{AP}-C}$$

We thereby enable explicit mutual authentication between legitimate C and AP. Our proposed solution additionally empowers one-sided anonymous identity validation for C. Further, after successful authentication process, AP and C also can set secured symmetric cryptosystem for future data exchange process. Each data exchange session will be solely identified by (${\mathrm{pid}}_{\mathrm{cj}}$, ${\mathrm{ID}}_{\mathrm{AP}}$).

## 4. Security and Performance Analysis

### 4.1. Security Analysis

We tackle the proposed system security level to verify whether the requirements mentioned in subsection security assumption have been satisfied. We will show how our scheme provides secure mutual authentication between C and AP, anonymity for C, leaked key security, unlinkability, and impersonation attack. Moreover, aggregated values in our proposed solution hide the contained accumulated individual records, which empower individual C’s data privacy protection. Recall the definition of the Decisional Bilinear Diffie–Hellman (DBDH) assumption in the random oracle model.

**Definition** **(DBDH assumption):** *The bilinear decisional Diffie–Hellman (*BDDH*) problem is defined in such a way that for known values* g, $g^{x},g^{y},{\;g}^{z}\;and$ *unknown random values* $\;x,\;y,\;z\in {R\;Z}_{P},\;\mathrm{and}\;T\in R,{\;G}_{T}$*, it is considered difficult to set* $T=e{\left(g,\;g\right)}^{\mathrm{xyz}}$ *from any random element in the target group. The (t, ϵ)*–BDDH *assumption is verified in G, if no t time algorithm has the probability of at least* $\frac{1}{2}+\epsilon$ *to solve the* BDDH *problem for non-negligible* ϵ.

♦ **Anonymity:** Each C gets a set of pseudo-identity ${\mathrm{pid}}_{\mathrm{cj}}\in {\mathrm{PID}}_{C}$ and its related partial secret key $S_{\mathrm{NM}}.H_{1}\left(S_{C}\right),$ uring registration process from NM. These pseudo-identities, rather than C’s real identity, provide strong privacy protection. Not any involved entity, not even AP, can identify C or recollect different transactions launched by the same C except NM. In practice, C sends a random message request $\mathrm{Req}=\left\{M_{C},\sigma_{C},V_{C}\right\}$ each time to AP. This message request contains secret values ($x_{C},\alpha$) and pseudo-ID ${\mathrm{pid}}_{\mathrm{cj}}$ that are random (not constant) values each time that C initiates an authentication process with AP. Only C can compute $V_{C}={\left(U_{C}\right)}^{\alpha }$ and $\sigma_{C}=H_{2}\left(M_{C}\right).S_{\mathrm{NM}}H_{1}\left(S_{C}\right)$ since these values require both secret values ($x_{C},\alpha$) and partial private keys $S_{\mathrm{NM}}H_{1}\left(S_{C}\right)$ for their calculation. Therefore, an attacker including NM, in order to compute $V_{C}$ must solve the inherited CDH problem; that is, he should perform $U_{C}=x_{C}\;P$ and then $V_{C}={\left(U_{C}\right)}^{\alpha }$ for unknown random secret values $x_{C}$, α which contradicts the CDH assumption. Therefore, C is anonymous and cannot be impersonated through our scheme. Therefore, our scheme guarantees data anonymity and identicalness (aggregated values) based on BDBH assumption in random oracle to resist chosen-plaintext attacks.
♦ **Mutual Authentication:** The client C’s signature $\sigma_{C}=H_{2}\left(M_{C}\right).S_{\mathrm{NM}}H_{1}\left(S_{C}\right)$ is in fact a signed based pseudo-identity. Therefore, it is impracticable to fake a genuine signature without prior access to the secret values $S_{C}=(r_{c}+H_{1}\left({\mathrm{pid}}_{\mathrm{cj}},{\mathrm{PK}}_{C},P_{C}\right)S_{\mathrm{NM}})\;\mathrm{mod})$ and $U_{C}=x_{C}\;P$ due to the NP-hard calculation complexity of the Diffie–Hellman assumption in $G_{1}$. Thereby it is very hard to deduce the partial private key $S_{\mathrm{NM}}H_{1}\left(S_{C}\right)$ using ${\mathrm{pid}}_{\mathrm{cj}}$, and ${\mathrm{PK}}_{\mathrm{NM}}$. Similarly, an attacker with no prior knowledge of AP’s partial private key $S_{\mathrm{NM}}.H_{1}\left(S_{\mathrm{AP}}\right)$ and secret values $U_{\mathrm{AP}}=x_{\mathrm{AP}}P$ and β cannot make a legitimate authentication code ${\mathrm{auth}}_{1}$. Further an adversary cannot compute ${\mathrm{auth}}_{2}$ and verify the equation ${\mathrm{auth}}_{2}={\mathrm{auth}}_{1}$ since he cannot solve CDH (definition 1) as described in the section above. Furthermore, only legitimate C and AP can compute $L_{C}=L_{\mathrm{AP}}=V_{C}.V_{\mathrm{AP}}$, due to the randomness and secrecy of $U_{C}$ and $U_{\mathrm{AP}}$ respectively. Therefore, a secured authentication process between C and AP is achieved by our scheme.
♦ **Unlinkability:** Recall that C uses different pseudo-identity ${\mathrm{pid}}_{\mathrm{cj}}\in {\mathrm{PID}}_{C}$ during each authentication process with an AP. Furthermore, only NM is aware of the relation between a given pseudo-identity and its original C’s identity. For that reason, excluding NM and C, no other entity is able to determine C or relate different authentication processes launched by the same C.
♦ **Leaked key security:** As described in Section 3, our scheme provides a random distinct session key each time an authentication process is initiated by C with AP. It is due to the randomness of the choice of secret values α$,\;\beta ,x_{\mathrm{AP}},x_{C}\;\epsilon {\;Z}_{q}{}^{*}$ by C and AP. Doing so, an attacker with a used key has a very slight chance to compromise succeeding sessions.
♦ **Impersonation attack:** To impersonate C or AP, an adversary should generate the correct values of ${\mathrm{auth}}_{1}$ and ${\mathrm{auth}}_{2}$, respectively, which is practically infeasible, as explained above (mutual authentication process section). Further an AP cannot generate a correct C’s signature $\sigma_{C}=H_{2}\left(M_{C}\right).S_{\mathrm{NM}}H_{1}\left(S_{C}\right)$ and $V_{C}$ in the message request, since he cannot access $S_{C}$ and $x_{C}$ otherwise the attack can be detected by C in verifying ${\mathrm{auth}}_{1}$. Likewise, an adversary that intercepts the message $M_{C}=\left({\mathrm{pid}}_{\mathrm{cj}}\;\left|\left|h_{C2}\right|\right|t_{1}\right)$ and tries to impersonate AP has a negligible chance of success due to the CDH assumption (mutual authentication process section) that is believed to be difficult. The performance analysis section highlights the security functional results comparison between our scheme and related works [7,8,9].
♦ **Data Aggregation:** Moreover, aggregated values in our proposed solution hide the accrued single value that enforces the privacy preservation of single C compared to related works [7,8,9]. To achieve this additional aggregated data feature, we designed a modified additively homomorphic IBE scheme from the Boneh–Franklin IBE cryptosystem [33]. The security proof lies on BDDH assumption in a random oracle (refer to security analysis section). This cryptosystem [33] is appropriate for our proposed solution (small sensing data reading) to achieve data aggregation and batch authentication. Our modified IBE scheme has four algorithms and we use $G_{1}$, $G_{2}$ of prime order q, P as generator of $G_{1}$, and a bilinear mapping e: $G_{1}$ × $G_{1}$ → $G_{2}$, such that $e\left(P^{a},Q^{b}\right)=e{\left(P,\;Q\right)}^{\mathrm{ab}}$, $\forall \;P,\;Q\in G_{1},\;\forall \;a,\;b\in {\mathbb{Z}}_{q}^{*}$, and e(P, Q) ≠ $1_{G_{2}}$ whenever $P,\;Q\in G_{1}$.

**Setup:** NM randomly picks as master private key (msk) a number $S_{\mathrm{NM}}\in Z_{q}{}^{*}$ and calculates its related public encryption key ${\mathrm{PK}}_{\mathrm{NM}}=S_{\mathrm{NM}}P$. Then NM chooses a hash function defined as $H_{1}:{\left\{0,1\right\}}^{l}\to G_{1}{}^{*},$ where the message space is $ℳ=\left\{0,\ldots ,\;l-1\right\}\subseteq Z_{q}{}^{*}$ with l=p(n)<q for some polynomial p and the cipher-text space is $C=G_{1}{}^{*}\times G_{2}.$

**Extract** (${\mathrm{PK}}_{\mathrm{NM}}$, msk, ${\mathrm{pid}}_{\mathrm{ci}}$): NM computes and sets $k=P^{S_{\mathrm{NM}}}$. Output ${\mathrm{SK}}_{{\mathrm{pid}}_{\mathrm{ci}}}=H_{1}{\left({\mathrm{pid}}_{\mathrm{ci}}\right)}^{S_{\mathrm{NM}}}\;\mathrm{and}\;k$.

**Enc** (${\mathrm{PK}}_{\mathrm{NM}}$, ${\mathrm{pid}}_{\mathrm{ci}}$, m). C randomly picks $b\;\in {\;Z}_{q}{}^{*}$; outputs $C_{{\mathrm{mpid}}_{\mathrm{ci}}=\;}\left(P^{b},P^{-m}.e{\left(H_{1}\left({\mathrm{pid}}_{\mathrm{ci}}\right),k\right)}^{b}\right)$.

**Dec** (${\mathrm{PK}}_{\mathrm{NM}}$,${\mathrm{SK}}_{{\mathrm{pid}}_{\mathrm{ci}}}$,$C_{{\mathrm{mpid}}_{\mathrm{ci}}}$). AP parses $C_{{\mathrm{mpid}}_{\mathrm{ci}}}\;\mathrm{as}\;\left(c_{1},c_{2}\right)$ and compute 

$m^{*}=c_{2}/e\;\left({\mathrm{SK}}_{{\mathrm{pid}}_{\mathrm{ci}}},c_{1}\right)$ and $m=\log_{\bar{P}}{\;m}^{*}$. The verification of our modified IBE lies on the fact that

$$\log_{\bar{P}}(m^{*})=\log_{\bar{P}}\left(c_{2}/e\;\left({\mathrm{SK}}_{{\mathrm{pid}}_{\mathrm{ci}}},c_{1}\right)\right)$$

$$\log_{\bar{P}}\left(m^{*}\right)=\log_{\bar{P}}\left(\frac{P^{-m}.e{\left(H_{1}\left({\mathrm{pid}}_{\mathrm{ci}}\right),k\right)}^{b}}{e\left(H_{1}{\left({\mathrm{pid}}_{\mathrm{ci}}\right)}^{S_{\mathrm{NM}}},P^{b}\right)}\right)$$

(4) $$\log_{\bar{P}}\left(m^{*}\right)=\log_{\bar{P}}\left(\frac{P^{-m}.e{\left(H_{1}\left({\mathrm{pid}}_{\mathrm{ci}}\right),P^{S_{\mathrm{NM}}}\right)}^{b}}{e\left(H_{1}{\left({\mathrm{pid}}_{\mathrm{ci}}\right)}^{S_{\mathrm{NM}}},P^{b}\right)}\right)=m$$

We prove that our proposed homomorphic cryptosystem is additive in message space by multiplying cipher texts:

$$C_{1}\times C_{2}=(P^{b}\times P^{b^{\prime }},{\;P}^{-m}.e{\left(H_{1}\left({\mathrm{pid}}_{\mathrm{ci}}\right),k\right)}^{b}\times P^{-m^{\prime }}.e{\left(H_{1}\left({\mathrm{pid}}_{\mathrm{ci}}\right),k\right)}^{b})$$

$$C_{1}\times C_{2}=(P^{b+b^{\prime }},{\;P}^{-m+m^{\prime }}.e{\left(H_{1}\left({\mathrm{pid}}_{\mathrm{ci}}\right),k\right)}^{b+b^{\prime }})$$

$$C_{1}\times C_{2}=\mathrm{Enc}\left({\mathrm{PK}}_{\mathrm{NM}},{\mathrm{pid}}_{\mathrm{ci}},m+m^{\prime }\;\mathrm{mod}\;q\right)$$

Note that the two disadvantages that come along with our modified additively homomorphic IBE scheme (i.e., the limited messages backup capacity and computing a discrete logarithm function to decrypt the data) are acceptable in many practical areas and especially in the e-Care system. Therefore, it does not affect the performance of our proposed solution. Table 2 shows clearly that our scheme is a good candidate to address the security shortcomings in the related works [7,8,9,29].

### 4.2. Performance Analysis

We describe our proposed solution performance analysis in comparison with related works [7,8,9]. First our scheme provides batch authentication between different client C and AP, which reduces efficiently the communication and computation cost. Upon receiving a gain access demand from C, AP checks the message’s signature authenticity in order to ascertain its related C (as described in Section 3). Further our scheme provides batch authentication, i.e., an AP can verify at the same time different message requests from various Cs securely through the help of NM. Thus, each $C_{i}$ sends its message requests $\left\{M_{\mathrm{Ci}},\sigma_{\mathrm{Ci}},V_{\mathrm{Ci}}\right\}$ to NM, which collects and forwards them as aggregated data to AP. Therefore upon receiving *n* distinct message requests denoted $\left\{M_{C1},\sigma_{C1},V_{C1}\right\},\left\{M_{C2},\sigma_{C2},V_{C2}\right\},\left\{M_{C3},\sigma_{C3},V_{C3}\right\},\ldots ,\left\{M_{\mathrm{Cn}},\sigma_{\mathrm{Cn}},V_{\mathrm{Cn}}\right\}$, respectively, from *n* different $C_{i}$ denoted as $C_{1},C_{2},C_{3},\ldots ,C_{n}$, with their respective signature $\sigma_{C1},\sigma_{C2},\sigma_{C3},\ldots ,\sigma_{\mathrm{Cn}}$, AP checks the correctness of this equation:

(5) $$e\left(\sum_{i=1}^{n}\sigma_{\mathrm{Ci}},P\right)\begin{array}{c} ?\\ = \end{array}e\left(\sum_{i=1}^{n}H_{2}\left(M_{\mathrm{Ci}}\right).H_{1}\left(S_{\mathrm{Ci}}\right),{\mathrm{PK}}_{\mathrm{NM}}\right).$$

**Verification**

$$e\left(\overset{n}{\underset{i=1}{\sum }}\sigma_{\mathrm{Ci}},P\right)=e\left(\overset{n}{\underset{i=1}{\sum }}H_{2}\left(M_{\mathrm{Ci}}\right).S_{\mathrm{NM}}H_{1}\left(S_{\mathrm{Ci}}\right),P\right)$$

$$e\left(\overset{n}{\underset{i=1}{\sum }}\sigma_{\mathrm{Ci}},P\right)=e\left(\overset{n}{\underset{i=1}{\sum }}H_{2}\left(M_{\mathrm{Ci}}\right).H_{1}\left(S_{\mathrm{Ci}}\right),S_{\mathrm{NM}}P\right)$$

$$e\left(\overset{n}{\underset{i=1}{\sum }}\sigma_{\mathrm{Ci}},P\right)=e\left(\overset{n}{\underset{i=1}{\sum }}H_{2}\left(M_{\mathrm{Ci}}\right).H_{1}\left(S_{\mathrm{Ci}}\right),{\mathrm{PK}}_{\mathrm{NM}}\right)$$

This data aggregation support in our model at the NM side has significant practical advantages for sensor networks. It facilitates efficiently keeping down the communicating cost between C and AP and empowers the privacy protection of a single $C_{i}$. Our proposed solution keeps down the number of transmitted data by sending one aggregated assessment (almost the size of a single report) instead of distinct individual message requests. Furthermore, this data aggregation feature hides the accrued single value, which enforces the privacy preservation of a single C compared to related works [7,8,9], and [29].

Note that the two disadvantages that come along with our modified additively homomorphic IBE scheme (i.e., the limited messages backup capacity and computing a discrete logarithm function to decrypt the data) are acceptable in many practical areas and especially in the e-Care system. Therefore, it does not affect the performance of our proposed solution (see Functioning Evaluation section). Based on this data aggregation and batch authentication support, the computing cost that AP needs to validate *n* signatures is largely composed of *n* point multiplications and two pairing calculations. Thus, the required time for AP to authenticate a large number of signatures from distinct C is obviously brought down. Therefore, it reduces the transmission loss proportion imputable to a possible bottleneck of digital signature authentication at the AP side. Recall that this batch verification operation has great merit for a limited power environment like WBAN.

For efficiency purposes, the multiprecision integer and rational arithmetic cryptographic library (MIRACL) [34] and cost-efficient pairing based cryptography (PBC) libraries are implemented into our proposed solution’s experiments to yield a 1024-bit security level. Experimental platforms are PCs with different computational power: Pentium(R) Dual-Core E6700 CPU 3.20 GHz, 4 GB RAM and 64-bit Intel®, 624 MHz processor, and 128MB memory to simulate AP and C, respectively. In the experiment, $G_{1}$ and $G_{2}$ are depicted by 160, 161, and 960 bits, respectively, and ${\mathrm{pid}}_{\mathrm{cj}}$, Timestamp, and ${\mathrm{ID}}_{\mathrm{AP}}$ by 32 bits. A Miyaji-Nakabayashi-Takano (MNT) curve is implemented with 160 bits, *k* = 6, depicting the order and embed degree, respectively, in $Z_{q}{}^{*}$. The performance evaluation is done based on related work experimental conclusions [8] depicted in Table 3. We focus on computations with expensive calculation costs, like modular exponentiation (TSM), ECSM (TSM), Hashing to point in $G_{1}$ (TGH) and bilinear pairing (TP) operations. Therefore, a computing time-based comparison study is done with the exiting related models as shown in Table 4.

Note that the computation cost for AP and C is one point multiplication for both two and one pairing calculations, respectively. Recall that the computing cost for a pairing function is much more expensive than a multiplication calculation. The client C may be a limited device; this low computation cost is a significant advantage for our scheme compared to the related work [7].

Based on Table 4 analysis, we can highlight our scheme efficiency on the obvious reduction of computation and communication costs for verifying *n* different signatures (batch authentication) from multiple clients by AP that consists of *n* point multiplications and two pairing calculations only. We also reduce the computation cost of C, which is a limited resource device in comparison to Wang and Zhang’s Model. This result is a desirable attribute for constrained power environments like WBAN.

## 5. Conclusions

This work presents a novel batch mutual authentication cryptosystem between WBAN’s controller/client C and an application provider AP. This proposed solution empowers the cryptosystem security level by providing batch authentication and data aggregation supports. We keep low the data transmission and computing over heads of C and AP using a lightweight ECC and efficient cryptographic pairing tools. Additionally, our solution needs only two handshakes between C and AP, without key certificate management like in the original asymmetric cryptography environment (PKI). Furthermore, our scheme efficiently provides an additive homomorphic IBE operation, in which a given AP can compute securely aggregated values from various WBAN clients. Our scheme reinforces privacy protection and reduces the running time on the client side. This is a great benefit for limited devices in environments like WBAN. However, we will improve the performance and security level by designing in our future work, a lightweight additive homomorphic IBE scheme with auxiliary input to address the side-channel attacks at the end user’s side.

## Figures and Tables

**Table 1:** Notations.

Symbols	Description
NM	Network manager
AP	Application provider
C	WBAN client/controller
q	Large prime number
$G_{1}$	Additive group with order q
$H_{i}$	Secure hash function with i = 1, 2
$s_{\mathrm{NM}}$	Network manager private key
E/Fq	Elliptic curve over prime field
Fq	Prime field
e || f	Concatenation of strings *e* and *f*
P	Generator of group G
${\mathrm{pid}}_{\mathrm{cj}}$	Client pseudo-ID, with j = 1, 2, 3
${\mathrm{ID}}_{\mathrm{AP}}$	Application identity
${\mathrm{PK}}_{\mathrm{NM}}$	Network manager public key

**Table 2:** Security comparison analysis.

Scheme	Wang and Zhang [7]	Liu [8]	Zhao [9]	Omala, A.A. et al. [29]	Our Scheme
Data aggregation	×	×	×	×	√
Mutual authentication	√	√	√	√	√
Anonymity	√	√	×	√	√
Impersonation attack	×	√	√	√	√
Unlinkability	√	×	√	√	√
Leaked key security	√	√	√	√	√
Batch authentication	×	×	×	×	√

**Table 3:** Cryptography running time operation based on results in [8].

	AP (ms)	C (ms)
*TME*	13.21	63.51
*TSM*	6.38	30.67
*TP*	20.04	96.35
*THG*	3.04	14.62

**Table 4:** Functioning Evaluation (Execution Time).

Schemes	AP (ms)	C (ms)
Wang and Zhang [7]	2*TSM* + 1*TP* ≈ 32.80	3*TSM* + 1*Tp* ≈ 188.36
Liu [8]	1*TME* + 1*TSM* + 1*Tpq* ≈ 39.63	1*TME* + 4*TSM* ≈ 186.19
Zhao [9]	6*TSM* ≈ 38.28	3*TSM* ≈ 92.01
**Our Scheme**	**1*TSM* + 2*TP* ≈ 46.46**	**1*TSM* + 1*Tp* ≈ 127.02**
